# *“Stabilise-reduce*, *stabilise-reduce”*: A survey of the common practices of deprescribing services and recommendations for future services

**DOI:** 10.1371/journal.pone.0282988

**Published:** 2023-03-15

**Authors:** Ruth E. Cooper, Michael Ashman, Jo Lomani, Joanna Moncrieff, Anne Guy, James Davies, Nicola Morant, Mark Horowitz

**Affiliations:** 1 NIHR Mental Health Policy Research Unit, Institute of Psychiatry, Psychology & Neuroscience, King’s College London, London, United Kingdom; 2 Faculty of Education, Health and Human Sciences, University of Greenwich, London, United Kingdom; 3 Department of Life Sciences, University of Roehampton, London, United Kingdom; 4 Independent Researcher, United Kingdom; 5 Research & Development Department, Goodmayes Hospital, North East London NHS Foundation Trust, Essex, United Kingdom; 6 Division of Psychiatry, University College London, London, United Kingdom; 7 All–Party Parliamentary Group for Prescribed Drug Dependence, Secretariat 2016–19, 2020-Present, Westminster, United Kingdom; 8 All–Party Parliamentary Group for Prescribed Drug Dependence, Secretariat 2015–19, 2020-Present, Westminster, United Kingdom; Keele University, UNITED KINGDOM

## Abstract

**Background:**

Public Health England recently called for the establishment of services to help people to safely stop prescribed drugs associated with dependence and withdrawal, including benzodiazepines, z-drugs, antidepressants, gabapentinoids and opioids. NICE identified a lack of knowledge about the best model for such service delivery. Therefore, we performed a global survey of existing deprescribing services to identify common practices and inform service development.

**Methods:**

We identified existing deprescribing services and interviewed key personnel in these services using an interview co-produced with researchers with lived experience of withdrawal. We summarised the common practices of the services and analysed the interviews using a rapid form of qualitative framework analysis.

**Results:**

Thirteen deprescribing services were included (8 UK, 5 from other countries). The common practices in the services were: gradual tapering of medications often over more than a year, and reductions made in a broadly hyperbolic manner (smaller reductions as total dose became lower). Reductions were individualised so that withdrawal symptoms remained tolerable, with the patient leading this decision-making in most services. Support and reassurance were provided throughout the process, sometimes by means of telephone support lines. Psychosocial support for the management of underlying conditions (e.g. CBT, counselling) were provided by the service or through referral. Lived experience was often embedded in services through founders, hiring criteria, peer support and sources of information to guide tapering.

**Conclusion:**

We found many common practices across existing deprescribing services around the world. We suggest that these ingredients are included in commissioning guidance of future services and suggest directions for further research to clarify best practice.

## Introduction

There is growing international concern about dependence on prescribed medicines, such as antidepressants, benzodiazepines, z-drugs and gabapentinoids, the difficulty some patients have in withdrawing from these drugs and a lack of appropriate services to support patients who have these difficulties [1–3] (see key definitions in Box 1). In the UK a recent Public Health England (PHE) Report identified the scale of the prescribing of drugs that can cause dependence and withdrawal as a significant public health issue [1]. It found that one in four adults in England were prescribed at least one prescription of a benzodiazepine, z-drug, gabapentinoid, opioid or antidepressant in 2017–2018, with up to a third of those prescribed for at least three years. It was estimated that the NHS may incur a loss of up to £564 million a year due to the unnecessary prescribing (i.e. non-indicated or dispensable) of dependency forming medications [4].

**Box 1. Key definitions**

**Dependence:** An adaptation to repeated exposure to some drugs and medicines usually characterised by tolerance and withdrawal. Dependence is an inevitable consequence of long-term use of some medicines and is distinguished here from addiction [1].

**Withdrawal:** Physiological reactions when a drug or medicine that has been taken repeatedly is removed [1].

**Bead counting:** Bead counting involves opening up capsules of medication which contain small time-release beads of medication–often hundreds per capsule. Patients will count these beads in order to make small reductions every fortnight or month.

**Tapering strips:** Medication can be compounded by a specialist pharmacy in order to make up smaller formulations of medication (e.g. 1mg or 5mg for medication commercially available only as 50mg or 100mg). Tapering strips are one example of compounded medication made by a specialist pharmacy in Holland in which increasingly small doses of medication are sealed in plastic strips [22].

This scale of prescribing and the importance of more effective deprescribing is an international issue. For example, antidepressant prescribing at levels comparable or higher to those in the UK, have been reported in Canada, USA, Australia, Portugal and Iceland [2, 3]. In the USA it is estimated that 10.4% of people are using benzodiazepines [5] and benzodiazepine-related deaths have risen [6], which has generated concern. The US Food and Drug Administration (FDA) updated a boxed warning for benzodiazepine medications to add information about the risks of abuse, misuse, addiction, physical dependence and withdrawal reactions [7]. In Norway due to dissatisfaction with aspects of mental healthcare, including high rates of prescribing of psychiatric medication, service user groups successfully campaigned for the introduction (from 2015) of medication-free mental health services into national policy. The medication-free services include specialist support for deprescribing psychiatric medications [8, 9]. Due to concerns around overprescribing and the need for more deprescribing support, a number of international and national deprescribing networks have been established for patients, carers, clinicians, and researchers, such as the International Institute for Psychiatric Drug Withdrawal [10], the US Deprescribing Research Network [11] and the Canadian Medication Appropriateness and Deprescribing Network [12].

Many people find it difficult to reduce or stop prescribed medicines of dependence due to experiencing withdrawal symptoms which can be severe and last for months, or years. Withdrawal symptoms can include anxiety, agitation, depressed mood, sudden changes in mood, muscle spasms, diarrhoea, dizziness, insomnia, nausea, brain ‘zaps’, flu-like symptoms, and suicidality [13, 14]. Protracted withdrawal symptoms can have a significant negative impact with loss of jobs, friends, homes and even suicidality [15, 16]. For example, antidepressant withdrawal is thought to affect 56% of people who attempt to stop these drugs, with up to 25% reporting withdrawal as severe [17]. Withdrawal symptoms can often resemble the symptoms of the condition for which the medication was initially prescribed and can be misdiagnosed as a recurrence leading to prolonged, potentially unnecessary treatment [17–19].

There is a lack of guidance, support and services to help people safely stop prescribed medicines of dependence [1]. This is pertinent in light of the Structured Medication Reviews mandated by the NHS to combat overprescription and polypharmacy which will identify more people who would benefit from deprescribing [20]. The PHE report recommended that: 1) local authorities should commission ‘tiered support’ services (e.g. a range of options) and 2) a helpline and website to help people safely withdraw from prescribed medicines of dependence [1]. The NHS intends to publish a commissioning framework for deprescribing services, but recent National Institute for Health and Care Excellence (NICE) guidance highlighted a lack of research on ‘what service models are most effective in supporting withdrawal’ from these medications [21].

Therefore, we conducted an international survey of deprescribing services with the aim of summarising current practice to guide deprescribing service development. Our research question was: ‘In deprescribing services what are the common practices to support patients to withdraw from prescribed medicines of dependence?’

## Methods

This was a rapid project due to funder requirements with project set-up, data collection and analysis restricted to 3 months (Jan-March 2021). A small amount of further funding was obtained to finalise the analysis and write the manuscript.

### Ethical approval

Ethical approval was not required as this project was a service evaluation (confirmed by the University of Roehampton Ethics Committee). Written informed consent was obtained from all participants.

### Research team and reflexivity

The multidisciplinary project team consisted of a consultant psychiatrist, a training psychiatrist, researchers, lived experience researchers, and psychotherapists. The project team brought relevant expertise in mixed methods and qualitative research methods, lived experience research and co-production and clinical approaches. All interviews were conducted by MA (lived experience researcher). One service (two interviews) did not wish to be recorded therefore MH (Clinical Research Fellow) also conducted these interviews to take notes.

### Survey

The survey consisted of 63 questions across 11 topics, with data collected through a structured interview (Supplement 1 Appendix in S1 File). The survey was co-produced by the lived experience researchers and multidisciplinary project team, based on: i) information on deprescribing services requested by the NHS as part of an evidence call [1]; ii) the TiDiER checklist (template for intervention description and replication) [23]; iii) our lived experience of medication withdrawal. The qualitative aspects of the study are reported according to the COREQ checklist (Consolidated Criteria for Reporting Qualitative Research) (Supplement 2 Appendix in S1 File) [24].

### Deprescribing service definition and recruitment

We included any service that met the following inclusion criteria: i) the service specifically aimed to support patients withdrawing from prescribed medicines of dependence, such as antidepressants, benzodiazepines, gabapentinoids, opioids, and z-drugs; ii) in which at least one staff member could interview in English. We excluded self-help and mutual-support groups.

We identified services through: i) our international deprescribing networks including the International Institute of Psychiatric Drug Withdrawal, which is the single global institution for researchers, clinicians and service users interested in safe deprescribing of psychiatric medications with extensive links to worldwide services [10] and the All Party Parliamentary Group for Prescribed Drug Dependence in the United Kingdom [25]; ii) the Public Health England report on dependence and withdrawal associated with prescribed medicines which included examples of deprescribing services following a national review of services [1], iii) through asking services to identify other services (snowball sampling). The services were initially approached via an email which included the study information sheet. All services we approached agreed to take part in the study and an online interview was then arranged.

### Data collection

For each service, at least one interview was conducted with a manager and/or other knowledgeable senior staff member, by a lived experience researcher via video call. Participants were provided with an information sheet and completed informed consent. The audio of the interviews were recorded. Field notes were made during and after the interview. One service did not consent to recording therefore notes were made by MH.

### Analysis

Service characteristics were summarised and reported using guidance from the TiDiER checklist [23]. A figure providing an overview of common deprescribing service features was developed and reviewed by the research team to reach consensus.

To explore the deprescribing services in detail, we conducted rapid qualitative framework analysis of the interview data using field notes and audio recordings with selected transcription [26–28]. The audio and field note data were augmented with information documents about the services and their websites to provide contextual understanding and triangulation of information about services [28].

Rapid qualitative analysis is an established methodology [28] chosen for our study given available resources, and the short timeframe—with the main project restricted to three months. We based our analysis on guidance on framework analysis [27], rapid qualitative analysis [28] and team-based co-production processes [29]. This ensured that analysis incorporated perspectives from the multidisciplinary research team, including those with lived experience [28]. Selected transcription is an established method in rapid qualitative work that enables analysis of data directly from audio recordings and is suitable for analyses focused on specific topics. It involves the researchers listening to the interviews in full and transcribing example quotes that address the research question, and illustrate relevant domains of the coding framework (see analysis phases below for further details) [28].

The rapid qualitative framework analysis occurred in four phases:

1. **Preliminary coding:** three research team members (MA, MH, RC), including those with lived experience of psychiatric medication withdrawal, independently listened to 2 deprescribing service’ recordings (in full). Key domains, emerging from the data, that addressed our research question, ‘In deprescribing services what are the common practices to support patients to withdraw from prescribed medicines of dependence?’, and articulated core aspects of the interviewee’s experience and understanding of the service were noted. The domains comprised of a label, brief definition and example quotes from selected transcription.
2. **Developing a coding framework:** i) the research team met twice to review and collate domains and develop a provisional coding framework (in Microsoft Excel). Similar domains were grouped together with example quotes selected. An ‘other’ category was kept for data that did not ‘fit’ the framework. ii) The whole team met to review and refine the framework. iii) The smaller research team then met to review and produce a working coding framework.
3. **Initial coding of data:** The research team coded the remaining withdrawal service recordings using the coding framework. Interviews were coded by listening to the recordings (in full) and transcribing example quotes under each domain. Researchers noted where data did not fit the framework into the ‘other’ category with new domains added to the framework as necessary.
4. **Refining the coding framework and writing up the analysis:** the research team met to discuss and refine the coding framework and resolve any coding uncertainties. The final framework was used to write up the analysis.

The manuscript was sent to participants who were asked to ensure that their service was accurately reported and were asked any outstanding questions about their service.

## Results

### Descriptions of services and common characteristics

**Fig 1:**
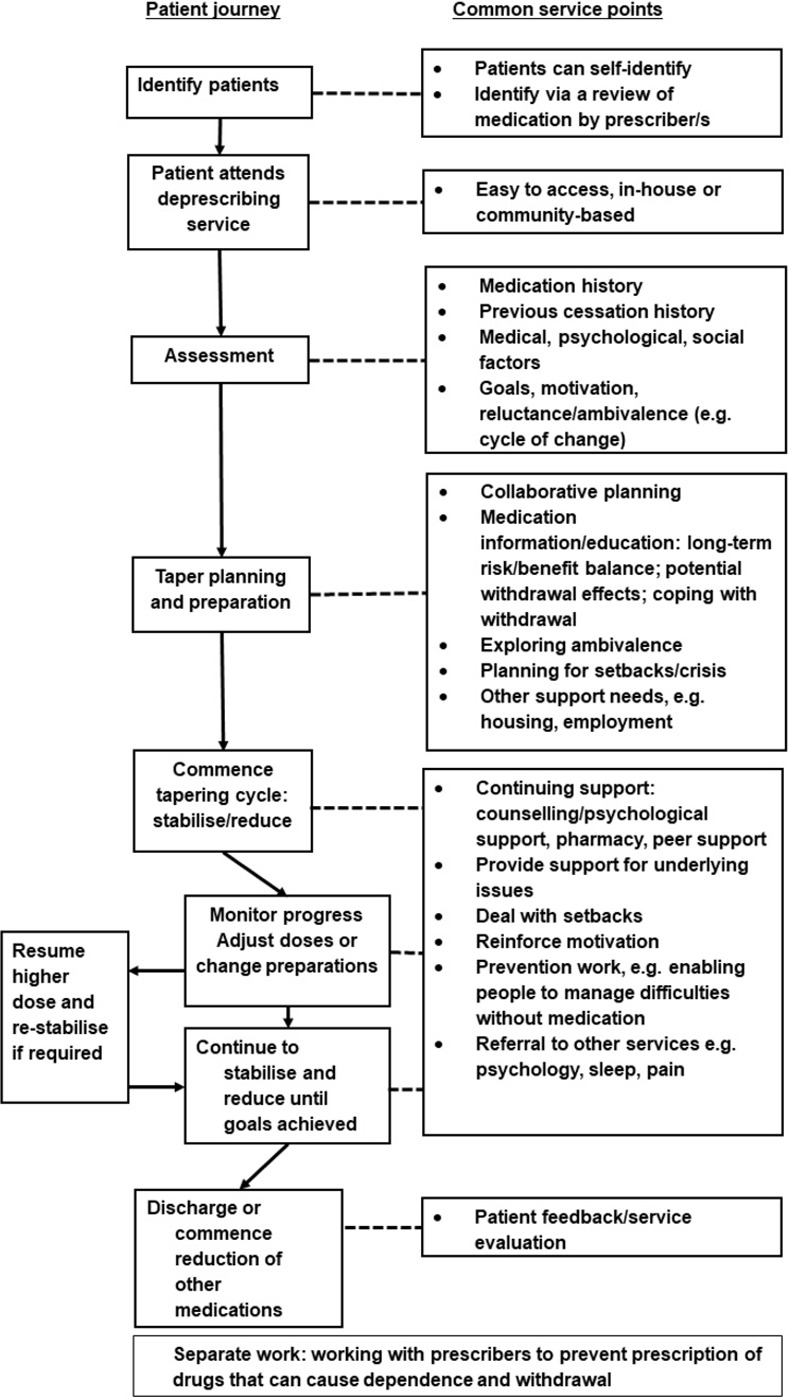
The patient journey and deprescribing service features.

We included 13 deprescribing services, 8 were UK-based, 1 was in the USA, 1 Norway, 1 Italy, 1 Sweden, and 1 Denmark. The services were based in primary or secondary healthcare, charities or private practices. We interviewed 18 participants, interviews lasted approximately 1 hour to 1 hour 45 minutes. Table 1 shows an overview of service characteristics, Table 2 shows tapering methods used in the deprescribing services. Fig 1 illustrates the patient journey and common service features. We discuss the common practices of deprescribing services. Further details and complexities are discussed in our qualitative findings, summarised with further example quotes in Table 3.

**Table 1:** Deprescribing services characteristics.

Service, interviewee	Setting, established	Target medications	Support offered	Funding	Mode of delivery	Clinical staffing mix	Referral sources	Cost per person helped if known (costing year)	Outcomes if known
**UK Services**									
S1 1 Manager	Within primary healthcare service, 2000	Benzodiazepines, Z-drugs, antidepressants, painkillers, "other" e.g. off-label antipsychotics	Medications information, taper support, counselling, peer support group, social/ psychological support^a^	State funding, free at point of contact	Face to face, phone, groupwork	Nurses, counsellors	Self, primary and secondary healthcare	£272 (2018)	33% cessation April-Sep 2018 15% cessation Oct-Dec 2019 18% cessation Apr-June 2020
S2 1 Manager	Charitable contractor, 1985	Antidepressants, benzodiazepines, Z-drugs, antipsychotics at GP request only	Medications information, taper support, national phoneline, counselling, weekly drop-in, peer support group, social/ psychological support^a^	State funding, free at point of contact	Face to face, phone, groupwork	Counsellors	Self but GPs will signpost	£333 (2018–19)	From the helpline 83% of clients commenced withdrawal, 67% of clients off their medication, 85% of clients reported a reduction in GP visits (2020 report)
S3 1 Recovery worker	Charitable contractor, 2008	Opioids, gabapentinoids, benzodiazepines, Z-drugs	Medications information, taper support, counselling, peer support—group and individual, social/ psychological support^a^	State funding, free at point of contact	Face to face, phone, groupwork	Drug worker/counsellors, lived experience volunteers	93% GP, 7% self	£269 (2016–17)	43.3% successful completions for benzodiazepines, 47.4% successful completions for opioids (2016–17)
S4 1 Recovery worker/manager	Charitable contractor, 1988	Benzodiazepines, Z-drugs	Medications information, taper support, counselling, peer support group, social/ psychological support^a^, yoga, GP advocacy^b^	State funding, free at point of contact	Phone, groupwork	Recovery worker, sessional group facilitator, volunteer counsellors	Self-referrals "vast majority", some referrals from parent organisation and primary and secondary care	£376 (2018)	Withdrew completely: 29% (2018)
S5 1 Nurse consultant	Within secondary healthcare service, 2019	Any psychotropic	Medications information, taper support, social/ psychological support^a^	State funding, free at point of contact	Face to face, phone	Nurse Consultants (NMP)	Medications review in secondary care wellbeing pathway	-	-
S6 1 Consultant psychiatrist	Within secondary healthcare service, 2020	Any psychotropic is planned but only antipsychotics and mood stabilisers to date	Medications information, taper support, social/ psychological support^a^^,^ peer support is planned.	State funding, free at point of contact	Face to face, phone	Consultant psychiatrist, pharmacist	GPs	-	-
S7 1 GP and specialty doctor in substance misuse, 3 Nurses, 1 GP/Academic	Based in substance misuse service in secondary care, (primary care liaison model), 2017	Benzodiazepines, opioids, Z-drugs, gabapentinoids	Medications information, taper support, social/ psychological support^a^ peer support groups, CBTi	State funding, free at point of contact	Face to face, phone	GP, substance misuse trained nurses	Self, GP and secondary care	-	2019/20: Of 209 clients, 25% completely stopped medication, 25% reduced their dose and were discharged, and 44% continued to reduce, 6% dropped out
S8 1 Consultant psychiatrist, 1 senior pharmacist	Within healthcare service, 2021	Any psychotropic	Medications information, taper support, social/ psychological support^a^^,^ peer support group and individual	State funding, free at point of contact	Face to face, phone	Consultant psychiatrist(s), pharmacist	Consultant psychiatrists	-	-
**Other countries**									
S9 USA 1 Consultant Psychiatrist/Academic	Private psychiatric practice, 2020	Any psychotropic	Medications information, taper support, social/ psychological support	Sessional payments	Face to face, phone	Consultant psychiatrist	Self	$150 per visit, up to 25 visits per year	-
S10 Norway 1 Consultant Psychiatrist	Within secondary healthcare service, 2017	Any psychotropic but antipsychotics are priority	Medications information, taper support, peer support, social/ psychological support in appointments and network meetings^a^, exercise, psychological and creative therapies, group and individual work for all strategies, social activities	State funding, free at point of contact	Face to face, groupwork	Consultant psychiatrist, mental health nurses, support workers, psychologist, art and physical therapists, paid Expert by Experience roles	Specialist mental health services refer but patients must request this	-	-
S11 Italy 1 Psychiatrist	University spinoff (effectively a private practice), 2018	Antidepressants, benzodiazepines	Medications information, taper support, CBT, well-being therapy, social/ psychological support^a^	Sessional payments	Face to face, phone	Consultant psychiatrists, psychologists, pharmacists	Self	-	-
S12 Sweden 1 Psychotherapist/Social Worker	Private practice within charitable foundation	Mostly antidepressants, some antipsychotics, will work with any psychotropic	Medications information, taper support, social/ psychological support^a^	Sessional payments	Face to face	Psychologist	Self	-	-
S13 Denmark 1 Clinical Psychologist	Private psychology practice, 2012	Any psychotropic	Medications information, taper support, social/ psychological support^a^	Sessional payments	Face to face, phone	Psychologist with lived experience	Self	-	-

a. Social/psychological support in appointments: general emotional, psychological, practical, and social support provided during regular appointments, e.g. reassurance about the time limited nature of withdrawal symptoms, coping strategies for withdrawal symptoms;

b. GP advocacy: negotiating directly with prescribers or supporting clients to negotiate with their prescribers in order to obtain GP support for medication withdrawal.

NMP: non-medical prescriber

**Table 2:** Tapering methods used in the deprescribing services.

Service	Typical taper duration	Frequency of contact	Tapering method	Switching medications	Dose reduction aids e.g. liquids, pill cutters
**UK Services**					
S1	12+ months	1–4 weeks depending on patient needs	Based on CITA^a^ guide [30]. Individualised, gradual, titrated to withdrawal effects, approximately 10% dose reductions every 2–4 weeks. Shared decision making^b^	Yes, but will try to remain on original medications, client wishes respected, will consult with GP or consultant psychiatrist before switching	Difficulty getting liquid preparations prescribed, do not recommend grinding, will use bead-counting
S2	12–24 months	Helpline clients call daily/weekly depending on needs. All face-to-face services are weekly.	Based on CITA^a^ guide [30]. Individualised, gradual, titrated to withdrawal effects, 10% dose reductions (calculated from most recent dose) every 4 weeks for benzodiazepines and every 6 weeks for antidepressants. Shared decision making^b^.	Will switch medications for half-life consideration	Will consider liquid preparations if available
S3	12+ months	Fortnightly Less complex clients: every 3 weeks or monthly Able to contact by text or phone in between appointments	Gradual, individualised, exponential, titrated to withdrawal effects, shared decision making	Will switch to diazepam with benzodiazepine reduction but will respect client preference if they do not wish this	Will consider liquid preparations if available
S4	12+ months	Weekly peer support group, phone and face to face check-ins	Use guidance from Ashton Manual [31]. Show patients and GPs relevant sections from NICE and BNF guidelines. Individualised, exponential, titrated to withdrawal effects, shared decision making^b^. Typically, 10–12.5% dose reduction of most recent dose (i.e. exponential) every 2–4 week intervals if stable after previous decrease	Will often write to GP to request switching medications.	People choose their own methods, e.g. pill cutters, grinding, requesting liquids from GPs
S5	1 month-3 years	Initially weekly to 2-weekly then needs-dependent	Individualised approach using small decrements at long intervals over long periods of time	Will switch medications, e.g. swap from olanzapine to aripiprazole (for side effect considerations rather than withdrawal). Doesn’t use half-life as a factor.	Tries to avoid pill-cutting/bead counting and uses available tablet doses (or vial sizes for depots, reduces effective depot doses by decreasing injection frequency). Uses liquids where GPs agree costs
S6	"Months or longer"	Monthly follow-up	Tapering is individualised, slow and cautious, other current medications taken into account. Typical withdrawal could be olanzapine reducing from 20mg in 2.5mg decrements with stabilisation between reductions. Aware of the need for additional caution in final low dose phases of withdrawal. Will go slower with smaller decrements in the later stages of reduction. Uses guidance on hyperbolic antidepressant and antipsychotic withdrawal [18, 32]	Has not done this so far but would consider changing medications in future with half-life as guide.	Pharmacist advises on what is appropriate to cut etc, does not "officially recommend" bead counting, uses liquids. Has not yet used dilution, ‘tapering strips’ not available.
S7	12–24 months	1–4 weekly follow-up	Service developed manualised method of slow reduction: gradual changes with reduced doses for increasing days each week. Slower in final stages. Schedule enforced (limited number of tablets given).	Will sometimes switch benzodiazepine clients to diazepam or opiates to morphine or reduce the medication the patient is already taking.	Liquids rarely used due to cost, will use available tablet doses mostly, pill-cutting in final stages depending on formulations available.
S8	Not known, new service	Monthly	Will use guidance on hyperbolic antidepressant withdrawal [18]. Trying to identify guidelines for other medications. Follow manufacturer’s guidance for e.g. lithium but this is "tailored to that patient and what else is going on for them"	Will consider switching preparations	Planning to use available dose decrements, pill cutters, dissolving tablets (depending on solubility) especially orodispersables, liquids/oral syringes "towards the end".
**Other countries**					
S9 USA	2–3 years	Initially weekly	States that "there is no specific guideline about how to do this" but will use guidance on hyperbolic antidepressant withdrawal [18], individualised to patient wishes and formulations available	Prefers to avoid this	Depends on patient wishes and formulations available, prescribes lower dose tablets, will use liquids, bead counting, jewellers’ scales, would like to use ‘tapering strips’ but these are not available to them.
S10 Norway	6 months-2 years	Constant—in inpatient setting	Use translated version of the ‘harm reduction guide to coming off psychiatric drugs’, i.e. exponential and slow [33]	Sometimes change drugs when tablet decrements are not available. Don’t switch to longer-acting medications.	Usually available tablet decrements, have used ‘tapering strips’ but these are no longer available, tablet cutting in later stages, usually switch depot patients to oral medications
S11 Italy	8–10 months	Initially every other week, thereafter once per month	Reductions every 2–3 weeks. Timescales depend on responses to early reductions which are closely monitored, especially for suicidal ideation, tapering schedule is individualised, accounting for previous withdrawal experiences	Will switch SSRI antidepressant to tricyclic if suicidality is seen as a withdrawal reaction, has in the past tried switching to longer acting formulations including fluoxetine (often with negative results), depending on reaction to withdrawal	Has used bead counting for venlafaxine. Mostly use available tablet decrements followed by liquids in the final stages of reduction.
S12 Sweden	2–3 years	Initially weekly, then reducing to monthly over time	Gradual slow reduction, reducing decrements in later stages, individualised but not explicitly "exponential"	Not known	Available tablet doses in early stages, final stages liquids, pill-cutting or grinding
S13 Denmark	12+ months	Unclear	Suggests people on multiple medications withdraw antidepressant first as antipsychotics will mask antidepressant withdrawal effects. Taper rate slows in final stages. Observed that antipsychotic withdrawal is more complex requiring additional caution in reduction rate	Will switch to preparations that can be more easily divided e.g. by cutting or dissolving, will also use half-life guided by pharmacists and Groot website.	Suggests liquid preparations if they are available, would use ‘tapering strips’ if these were available, pill cutters, dissolving.

a = CITA: Council for Involuntary Tranquiliser Addiction guidance: Individualised, gradual, titrated to withdrawal effects, approx 10% dose reductions every 2–4 weeks (e.g. diazepam >20mg,: 2mg reduction; 4-20mg: 1mg reductions; <4mg: <1mg reductions);

b. patient decision about reducing made with advice from service.

**Table 3:** Qualitative domains.

Domain	Key points	Illustrative quotes
**1. The patient journey** This domain describes how within the patient journey process several service features contribute to withdrawal and provide critical augmentation to the process of tapering.	Intake and assessment	*“…typically they have tried to taper off the drugs themselves and have had a lot of trouble doing that*.*”* (S9) *“We have a very web-oriented perspective on treatment… the hospital*, *the family and then maybe the workplace and then a friend or the school or whatever…it’s a very collaborative approach…”* (S10)
	Planning and preparation	*“…we talk about what it might feel like* [to reduce] *and things that we can do to alleviate that anxiety… basic kind of anxiety-management techniques… we will sometimes have a conversation about just pure breathing techniques*, *really good sleep hygiene*, *caffeine reduction*.*”* (S5) *“We will generally go through what people have experienced as useful or helpful in how they have dealt with things* [during any prior withdrawals] *and try to see if there are things within those kind of experiences that can be strengthened*.*”* (S10)
**2: The complexities of tapering** This illustrates the complex demands on withdrawal support, and why these have led to the service features within the patient journey shown in Fig 1.	Range of patient views on reducing medication	*“…people typically come in with a very specific idea that I wanna taper off these Medications and I wanna start doing it right away"* (S9) *“…there is that integral fear* [of relapse], *and it will have been quite clearly messaged to a lot of them*, *that if you don’t stay on this medication this will happen again*.*”* (S5)
	Lack of official guidance on withdrawal.	*“…there is no real clear withdrawal process*, *there is no specific guideline about how to do this*.*”* (S9) *“…what might fit one person won’t fit somebody else*. *There’s no formula or schedule*.*”* (S2)
	Distinguishing withdrawal and relapse	*"if you just take someone’s medication down by 5 milligrams and do not have a conversation about the possibility of anxiety and feeling unsettled then of course they will think ’oh my god*, *something terrible is happening*, *I’m reducing my medication and I’m becoming ill again"* (S5) *“…sometimes suicidal ideation can be really severe as part of withdrawal syndrome*.*”* (S11)
	Managing the responses to withdrawal	*“There are other cases in which each time you decrease the dose you start to have suicidal ideation and in this case we must go slower in order not to have the person constantly at risk of severe symptoms*.*”* (S11) *"usually the biggest reassurance is that if they are really struggling and they’re not happy and they’re not feeling well is that we always have a contingency to take it back up again…and it might be in the initial increment or it might be in a smaller increment but I always advise or reassure people that if they don’t manage it this time*, *then have a break and we might try it again another time"* (S5)
	Smaller reductions at lower dosages	*“We go down with the pills until we reach a certain dosage*, *usually this dosage shows to be extremely problematic in going ahead using pills*.*”* (S11) *“Everything* [to achieve low doses]…*bead counting*, *pill cutter*, *dissolving all that kind*, *some people are on the*…*ones you can’t cut*…*sometimes you have to change* [tablets]” (S13)
	Managing without medication	*“If somebody has been on a benzo then sometimes you find it’s been covering up something like sexual abuse or something else so you might refer on to specialist services…”* (S1) *"advocacy would come from me and that’s around principally the prescribing*, *and also probably second*, *maybe*, *housing…personal independence payments* [a UK welfare benefit], *that kind of thing comes up very very regularly and as part of the person’s sort of recovery kind of healing package it’s maybe hard to start a benzodiazepine taper if you haven’t got somewhere to live or money struggles"* (S4)
**3. The role of lived experience in withdrawal support** This sets out the role of lived experience in developing knowledge, methods and peer support in deprescribing services	Peer support	“There obviously will be peer support. If someone is finding something difficult then someone else in the group will say well have you tried this? Or I had that symptom.” (S2) “…well a lot of people say it’s really impactful and they get so much out of that particular modality, that way of, you know, mutual aid, listening to people’s stories, getting people to support one another.” (S4)
	Lived experience knowledge	*“I have learnt a lot from Adele Framer who runs Surviving Antidepressants…and honestly I’ve learned a lot from the patients themselves*.*”* (S9) *“…everything that I’ve described to you is what patients have told me and what I’ve observed with patients*.*”* (S5)
**4. Outcomes** This shows complexity within outcomes as seen in deprescribing services.	Qualitative outcomes	*“…getting better*, *in a nutshell it’s when all the symptoms have gone*, *they’re feeling better and they’re back to their normal selves*, *who they were before they started taking the medication… it’s coming off completely… complete cessation*.*”* (S2) *“I have met also people who have really*, *really tried to get off antipsychotics*, *for instance*, *and have decided to keep a small dose because as they say themselves it’s too hard to get off all of it*.*”* (S12)
	Prevention	*“…a successful outcome is ultimately not to need secondary mental healthcare any more*, *secondly being in a place where they are really happy and satisfied with physically and mentally*.*”* (S5) *"a successful outcome for us would can be the patient gets off medication and still has some withdrawal symptoms but knows how to manage them and*, *let’s say*, *de-activated that kind of psychological mechanism which in other times would drive them to ask for a prescription”* (S11)

#### Service development

The services were established by healthcare professionals or former patients. Reasons for service development included: i) concerns around prescribing practices, e.g. high levels of prescribing, polypharmacy, and limited knowledge about withdrawal, such as the mis-interpretation of withdrawal symptoms as relapse of an underlying condition; ii) lived experience of withdrawal and identification of an absence of withdrawal support; iii) providing an alternative to addiction services.

#### Medications

Most services worked with patients prescribed a range of psychotropic medications, including benzodiazepines, antidepressants, z-drugs, opioids, and gabapentinoids, with some addressing antipsychotics. Four services targeted specific medications: benzodiazepines, z-drugs (S4), benzodiazepines (S7), antidepressants, benzodiazepines (S11), and one focused on antipsychotics, but included other psychotropics (S10).

#### Referrals

Referrals to services came via different routes, e.g. self-referral, primary or secondary care, medicines optimisation programmes.

#### Intake and assessment

Assessment at intake included: current prescription(s), reason for prescription, medication history, current medical, social and psychological circumstances, beliefs about medications, previous cessation attempts, reasons for stopping and facilitators and barriers to stopping.

#### Tapering planning and preparation

At the planning stage a tailored reduction programme is agreed with regular dialogue and monitoring appointments.

#### Tapering guidance

The following tapering strategies were commonly reported by services (more detail in Table 2):

*Tapering medications gradually using broadly hyperbolic strategies*. Most services tapered medications over at least a year or longer. Doses were reduced at 2–6 week intervals, allowing patients to stabilise before the next reduction. Taper lengths generally ranged from 6 months-3 years. Some services used broadly hyperbolic strategies meaning that the steps by which the dose is lowered are made smaller and smaller as the dose decreases, e.g. reductions of 10% of the prior dose.

*Individualised*, *flexible tapering*, *using shared decision making*. All services would monitor for withdrawal effects and adjust the taper rate accordingly. Shared decision making was prioritised by most services, in that the decision when and how to reduce was made by the patient with the advice of the service.

*Tapering guidance*. Services used a range of tapering guidance, e.g. CITA (Council for Involuntary Tranquiliser Addiction) guidance [30], an academic paper [18], and the ‘Harm Reduction Guide to coming off Psychiatric Drugs’ [33].

#### Psychosocial support–monitoring

All services provided psychosocial support (see Table 1). The majority of psychosocial support was non-specific during regular monitoring appointments. During monitoring appointments, services would assess tolerability to reductions, deal with setbacks and adjust doses and reduction intervals accordingly. Monitoring appointments would also provide emotional and practical support, e.g. reassurance about the time-limited nature of withdrawal symptoms, reinforcing strategies for dealing with withdrawal effects such as anxiety management, sleep hygiene, and exercise. Some services also encouraged patients to diarise withdrawal effects.

#### Additional psychosocial support

Services also provided (through onwards referrals or by the services) additional psychosocial support, e.g. counselling, Cognitive Behavioural Therapy (CBT) (including for insomnia), peer support in 1:1 and group settings, and a phone line providing advice on reducing/stopping medications.

#### Outcomes

Only five (UK) services reported outcomes. For those reporting withdrawal rates over 1 year: 25%-67% of clients discontinued medications. For example, at one service (S7), 209 patients were assessed in 2019/2020, of these patients, 25% stopped their medication, 25% reduced their dose and were discharged, and 44% were continuing to reduce. However, no services provided data that tracked patients long-term, therefore reported figures may not reflect ultimate cessation/reduction levels.

### Qualitative findings

#### 1. The patient journey

All services provided a holistic approach to supporting withdrawal and stressed the importance of supporting people with more than just tapering medication. This approach often included finding ways to deal with any underlying issues, working with families and wider social networks, managing setbacks or crises and helping people cope with withdrawal and providing psychological or social support. Fig 1 is an amalgam of the reported patient journeys and is broadly followed by all services.

*Intake and assessment*. At intake, respondents described how, when patients self-referred this could be due to the reluctance of their prescribers to withdraw medication, with patients seen as *“not compliant”* (S10) if they wanted to stop medication. In cases of patient and prescriber disagreement, some services took over as prescribers, some negotiated with prescribers or advocated for patients, others may recommend changing prescriber. Some services also highlighted the importance of having dedicated deprescribing services for this patient group who were taking medication as prescribed by their clinician and the inappropriateness of such patients being sent to addiction services when misuse of medication was not relevant to this patient group.

Service providers discussed the importance of understanding patients’ medication beliefs and how these arose: some patients just want to discontinue, some believe symptoms will return or relapse may occur. These views may stem from what prescribers have told them, from patient networks and/or the internet, or from personal experience, including negative experiences from previous discontinuation attempts.

“*I don’t even touch the medications for the first 2 or 3 visits*, *I focus more on getting to know the person a little*, *understanding what has happened because of the medications*, *and what is possibly not because of the medications and how the meds are sort of woven into their own psychological context*.*”* (S9)

Assessments included patients’ social circumstances, which were facilitators or barriers to reduction. Practical difficulties such as housing or financial difficulties can hinder tapering:

*“it’s maybe hard to start a benzodiazepine taper if you haven’t got somewhere to live or money struggles*.*"* (S4)

Support networks both formal (e.g. clinical services) and informal (e.g. social networks) were assessed and contributed to planning and preparing a holistic reduction programme. For example, families were often supportive, although there could be conflict between patient and family views on discontinuation.

*Planning and preparation*. At the planning stage, for those reducing multiple medications, priorities about which to reduce would be discussed, with one medication reduced at a time. Services explored and strengthened what people found helpful in previous withdrawal attempts. All services reported that patients could contact them outside of routine appointments, e.g. through phone helplines. Patients were advised on what to expect during withdrawal, that the reduction would be flexible, on ways to cope with withdrawal symptoms, and reassured that support would be available at all stages. Reassurance of the transitory nature of withdrawal symptoms was seen as helpful, as was support for self-management of anxiety or problems with sleep.

*"I think a big part of withdrawal is dealing with all the affect that comes up*, *you’re going to get anxious*, *depressed*, *even desperate at times so we do a lot of preparatory work*, *in that sense*, *so they know that this may happen*, *I try to initiate some sort of…small lifestyle change before we start tapering*, *like starting to exercise a little bit*.*”* (S9)

#### 2. The complexities of tapering

There were a range of circumstances and challenges which affected the process of tapering.

*Range of patient views on reducing medication*. Patient attitudes to reducing medication ranged from significant desire to stop to ambivalence, reluctance, or fear. To support informed choice about reducing medications all services provide information on medication risks/benefits. Techniques such as motivational interviewing were perceived to enable compassionate and respectful exploration of ambivalence or reluctance towards medication reduction:

*“obviously if they really don’t want to do it then there’s nothing we can do but we do try to motivate the unmotivated*, *we do say well we can explain to you how the drug effects the body and we work on all of that*, *see if we can find a chink in the armour*, *see if they will consider even the tiniest reductions”* (S1)

Challenging medication beliefs also occurred, such as that a chemical imbalance is ‘corrected’ by the medication:

*“People still are very much told that they need*, *for instance*, *antidepressants because there is some imbalance in their brain…and then I typically used to say that the imbalance theory is very much of a myth which actually has not been possible to…validate*.*”* (S12)

Patients often had concerns about withdrawal symptoms, were fearful of relapse, believed that their original difficulties may return without medication, or worried that the medication may have *‘damaged’* them. Some people worried about relatives’ reactions to their decision to reduce.

*Interviewer*. “*What are people’s main worries when they start reducing their medication*?*”*

*Respondent*. “*I find definitely the sleep*, *they’re not going to be able to sleep which means that’s gonna wreck them for the next day*, *and if they’re in work they just feel that they can’t take that risk financially*, *that they’re gonna lose their job…*, *err… gut-wrenching fear and anxiety*, *the misunderstanding from friends and family on why they’re doing that when the doctor told them that they should be taking it*, *they’re not*, *maybe*, *being compliant with their treatment if there’s more of a psychiatric history*… *definitely the withdrawal effects themselves…”* (S4)

*Lack of official guidance on withdrawal and lived experience knowledge*. There is a lack of official guidance on tapering, and variability in the severity of withdrawal symptoms. Therefore, lived experience knowledge of withdrawal, from staff with lived experience, patients, or from patient led guidelines or websites, contributed to withdrawal guidance to patients and to educating service staff:

*“We use a lot of the knowledge*, *the user organisations’ own knowledge*, *for example the pamphlet by Will Hall* [the ‘Harm Reduction Guide to coming off Psychiatric Drugs’]*…we will generally tell people*, *how to taper…it’s not a scientific project*, *there’s not been done a lot of research around it*, *so the experience of how other people taper is basically what we have*, *so we base our tapering on these experiences”*. (S10)

Given this lack of guidance, the approach taken to withdrawal by all services was cautious, slow and individualised. At each reduction interval people were given time to adjust to the new dose and the impact of each reduction was assessed before proceeding to the next, summarised as:

*“stabilise-reduce*, *stabilise-reduce”*. (S4)

*Distinguishing withdrawal and relapse*. Respondents discussed how patients would experience a spectrum of withdrawal symptoms and how supporting tapering entailed difficulties in distinguishing withdrawal and relapse. Some respondents noted how withdrawal symptoms were sometimes similar to patient’s original difficulties:

*“Often discontinuation symptoms can be confused with re-emergence of symptoms*. *It can be really difficult clinically to work out what is going on*.*”* (S8)

Many patients were not well-informed about the occurrence and nature of withdrawal, and attributed symptoms that arose during reduction of medication to relapse of their previous problem.

*“The overwhelming idea is that if people have difficulties in reducing medication it’s a sign of a relapse and the need for the medication to be continued*, *I don’t think the idea of it being withdrawal symptoms is particularly well established*, *that’s something that I try to introduce*.*”* (S6)

*Managing the responses to withdrawal*. Responses to withdrawal symptoms involved adjusting dose reduction amounts or intervals, pausing the taper, or returning to the previous dose and re-stabilising. This enabled further monitoring of symptoms and an opportunity for discussion of how to proceed based on shared decision making. Patients would be reassured about the transitory nature of withdrawal symptoms and supported with self-management of e.g. anxiety or sleep. Counselling and psychological therapies could also be provided in the service or through onwards referral if specific issues arose.

*“I often use for example ’is it tolerable*? *Is it bearable*?*’* [the withdrawal effects], *because my experience is if people know this is a side-effect that hopefully will be temporary*, *then we can power through*, *now if it’s too intolerable I say go back up to the dose you were on previously and then we go slower"*(S13)

*Smaller reductions at lower dosages*. Many respondents described the difficulties experienced by people in the final stages of tapering and stressed the need to make smaller reductions at lower doses: *“the lower you go the harder it gets”* (S4). Such small reductions are practically difficult because of the limited availability of low dose formulations. Services reported a range of strategies to achieve these small doses, including using liquid preparations, tapering strips, pill cutters, and milli-or microgram-accurate weighing scales.

*“As the dose gets lower*, *I try to space out the time between subsequent dose reductions and also the amount that’s being reduced drops…when the doses go lower that’s when we start running into problems because we have to be really creative in coming up with different dosages and formulations*.*”* (S9)

*Managing without medication*. Across all services an important part of appointments was to support people to manage any re-emergence of the original issues which medication may have suppressed, such as, traumatic memories, pain or anxiety, in order to help people manage without medication:

“When they get to trust you like any therapeutic alliance they’ll give you more information and then the trauma starts coming through and we’d work on that as we’re reducing down, and as we reduce down, because they’re not self-medicating, as it comes to the back end it starts coming up more, and then there’d be big level of, it’s not even aftercare, that care…once they’re opiate or benzo free they’re clear-headed and we can start really working on what’s going on for them” (S3)

Counselling and psychological therapies would be provided to patients which were thought to enable people to be more in control, helping them to cope with withdrawal, and manage any underlying issues. Ultimately, they aimed to enable patients to utilise internalised coping strategies instead of medication. Some services would provide practical support, either internally or through referrals, such as with housing or benefits. Services also recognised that some patients may experience protracted withdrawal after discontinuation and would provide patients with ongoing support after stopping medication.

*“…the importance of not just necessarily taking away a treatment but replacing it or at least supporting any reduction with additional input whether that’s psychological or other*.*”* (S6)

#### 3. The role of lived experience in withdrawal support

All services reported the involvement of people with lived experience of withdrawal as vital. This included lived experience knowledge of withdrawal (see above), founding and/or leading services, having oversight (e.g. as trustees), working as experts by experience, or staff members with lived experience, volunteering in the services, or in one case campaigning to change government policy so that drug-free services could be established. Some services encouraged those with lived experience to talk about their experience to patients. In a number of cases, service job adverts would specify the need for lived experience of withdrawal.

Most services included peer support sessions. Peer support enables people to share knowledge, experiences, provide support, and listen to each other. Involving people who have successfully stopped their medication gives patients hope that they can manage to stop their medications:

*“…if I’ve got a client who’s struggling*, *which I do quite often*, *I’ll ask them* [peer volunteers] *if they’ll join us in a Zoom session to give their experience*, *people want to hear it from the horse’s mouth*, *as it were*, *rather than some professional*, *it can sort of motivate them to make changes…*.*it works really well*.*”* (S3)

#### 4. Outcomes

Respondents described a successful outcome for patients as: complete medication cessation or reduction to the lowest dose if cessation is not possible; reducing the number of medications taken by the patient, patient capacity to manage difficulties without or with reduced medication, and improved quality of life.

In keeping with the patient-centred approach of services, outcomes were seen as subjective to each patient. Although some practitioners saw complete cessation and a return to the premedicated self as the ideal, patients’ wishes to retain a low dose were respected, perhaps reflecting the difficulties experienced in tapering at extremely low doses. It was also recognised that some patients may experience protracted withdrawal after discontinuing medication, underlining the desirability of equipping people with coping strategies for withdrawal effects. Quality of life was seen as important and that patients were comfortable regardless of medication dose.

*“What I envision is that whatever doses the patient is taking or not taking they feel like themselves*, *they feel comfortable with where they’re at*.*”* (S9)

A further outcome was described by some respondents as the patient having strategies to manage any life stressors, psychological difficulties and any ongoing withdrawal symptoms, once they are off the medication, e.g. through receiving psychological therapy. This would prevent future difficulties with dependence forming medication, aiming to have *“de-activated that kind of psychological mechanism which in other times would drive them to ask for a prescription”* (S11).

## Discussion

We conducted the first international survey of deprescribing services to summarise common practices and guide future service development. We included 13 deprescribing services (8 UK based) which support patients to withdraw from a range of psychotropic medications. Given the recommendation by Public Health England for the commissioning of services to help people safely withdraw from prescribed medicines of dependence [1] and therefore the pressing need for more specialist support for medication deprescribing, our identification of only 8 UK deprescribing services, most existing outside the public health system (the NHS), highlights the lack of deprescribing services in the UK.

The most common practices in the deprescribing services were: i) tapering medications gradually, often over a year or longer, monitoring for withdrawal effects and reducing the speed of the taper at lower doses; ii) prioritising patient preference around tapering decisions and using a flexible, individualised approach to tapering; iii) incorporating lived experience leadership and knowledge into services, through e.g. peer support interventions; iv) providing psychosocial support for patients such as psychological therapy, emotional support, and coping strategies for withdrawal symptoms. Box 2 shows the recommendations for deprescribing services (developed from this research), policy implications and research recommendations.

**Box 2. Recommendations for deprescribing services, policy implications and future directions**

**Recommendations for deprescribing services**

This survey of deprescribing services has given the following overall recommendations for future services:

- **Drugs should be tapered gradually using hyperbolic strategies**. This means that the steps by which the dose is lowered are made smaller and smaller as the dose decreases, for example reducing the medication by 10% of the prior dose. To facilitate this, services will need to have access to small doses of medication such as liquids or use flexible techniques such as bead counting. Services routinely reported that patients on long-term medication often required more than a year to stop, with some patients requiring considerably longer.
- **Tapering should be individualised, flexible and use shared decision making.** Variation between individuals in physiological withdrawal symptoms required personalised care. Encouragement and support to continue was helpful, but coercion was generally not employed.
- **Psychosocial support should be provided to patients during and, if required, after withdrawal.** Following withdrawal the service could refer patients for further psychosocial support if needed. Psychological therapists may find the guidance on working with patients who are taking or withdrawing from psychiatric drugs, to be useful [55].
- **Lived experience should be integrated at all levels:** people with lived experience of successfully and unsuccessfully withdrawing from prescribed drugs of dependence need to be involved in the conception, development and running of services. To enable this in the UK, a Lived and professional Experience Advisory Panel for prescribed drug dependence (https://leap4pdd.org/) has been established.
- **The wider context of a patient’s life should be taken into account.** This could include inviting family or the social network to withdrawal appointments.
- **Dedicated deprescribing services for prescribed medications of dependence are necessary** and should be separate to addiction services as most patients find this inappropriate given that they are taking medication as prescribed.

**Embedded training and outreach programmes should be integrated** to promote joined-up care between deprescribing services and other health and social care providers.

**Implications in a UK policy context**

- **The roll out of the Structured Medication Review (SMR) programme will see increasing numbers of patients encouraged to reduce or withdraw from prescribed drugs by clinical pharmacists.** The programme guidance makes no reference to the possibility of withdrawal responses, tapering or the importance of support for those who might benefit from deprescribing [20].
- **The National Overprescribing Review (NOR)** [56] makes various recommendations aimed at reducing overprescribing but again does not acknowledge the need for withdrawal support.
- **Two new roles, the National Clinical Director for Prescribing (NHS) and the Patient Safety Commissioner (Government)**, recognise the need for stronger management of prescribing culture and advocacy for patients in the health system respectively, and are welcomed as positive developments.
- **The NHSE&I Commissioning Framework** (’Framework for action: Optimising personalised care for patients at risk of, or experiencing, prescribed drug dependence or withdrawal’ August 2022). This is due to be soon published, we will review this framework and make recommendations to the NHSE&I on the basis of the results of this study.

**Future directions and research recommendations**

We recommend the following further research is needed, in line with recent recommendations from NICE [21]:

- Evaluation studies of withdrawal services compared to care as usual in primary care. The study would be either a randomised controlled trial or a longitudinal cohort study with a control group. A long follow-up period of at least 2 years would be required.
- Quantitative research looking at how to adapt rate of withdrawal to an individual’s characteristics. Relevant characteristics could include type of drug used, length of use, dose of use, previous attempts at stopping, length of taper, provision of psychosocial support.
- Evaluation of different medication formulations for facilitating gradual tapers, including ‘tapering strips’ and liquid versions of medications.
- Lived-experience led qualitative research to explore and understand service-user experiences of withdrawal and deprescribing practices.
- Qualitative research to explore clinician attitudes to and understandings of psychotropic medication and withdrawal.

We found important details and complexities to the services through our qualitative findings. The importance of specialised deprescribing services was emphasised by the reluctance of some prescribers to reduce medication in response to patient request with patients viewed as non-compliant if they ask to reduce. A holistic approach to withdrawal was provided by services. Assessment and preparation for withdrawal were important and respondents described a range of attitudes from patients towards withdrawal, from being desperate to stop to fear or reluctance. Services would get to know the patient, provide information about the medication risks/benefits, understand what the medication means for them and why they want to stop. They would assess facilitators and barriers to reduction including their social circumstances and through exploring what had been helpful in prior withdrawal attempts. Services would also challenge beliefs that might perpetuate unnecessary use of medication, such as the ‘chemical imbalance’ theory. They would talk to patients about what to expect during withdrawal and provide reassurance to patients about the availability of support.

Once withdrawing from the medication respondents described the complexity and difficulty of distinguishing withdrawal from relapse. Patients often experienced a re-emergence of the original issues which the medication may have suppressed, such as traumatic memories or anxiety. The provision of counselling and psychological therapies was important in helping people develop internal coping strategies to help manage these difficulties, instead of medication. Peer support was also incorporated into services, giving patients hope that they would also be able to stop their medications. Some services pointed out that sending patients who were taking medications as prescribed to addiction services was inappropriate and often counter-productive and there was a need for specific deprescribing services for this patient group. Difficulties could occur in the final stages of tapering due to the limited availability of low dose formulations. The subjectivity of a ‘successful’ outcome was discussed by services which could be complete medication cessation or reduction to the lowest dose. Quality of life was prioritised along with ‘patient empowerment’, the patient being able to manage without medication in the future.

The services highlighted the relative absence of guidelines to support withdrawal from prescribed medicines of dependence [1, 19, 34]. Therefore, guidance was drawn from lived experience, informal guidance (e.g. [31]) and clinical experience and taking a slow and cautious approach to withdrawal was vital. However NICE has recently published guidance on medication withdrawal [21], a considerable step forward, although this guidance has been criticised for a lack of practical detail making implementation challenging [35].

Although outcome data were limited to 5 services and therefore may not be representative, of those reporting 1 year outcomes, 25%-67% of patients were able to stop their medications. This is higher than discontinuation rates in studies in primary care in which antidepressant discontinuation rates may be as low as 6–7% [36, 37], suggesting considerable value to deprescribing services.

Tapering medications gradually and slower at lower dosages, is in line with the existing limited evidence and guidance on withdrawal strategies for a range of medications, including antidepressants, benzodiazepines, antipsychotics, opioids, and z-drugs [18, 21, 32, 38–41]. A systematic review found one trial demonstrated a 6-fold increased chance of stopping benzodiazepines for gradual dose reduction compared with routine care [42]. Such reviews demonstrate that gradual tapering is superior to abrupt cessation but they lack the necessary granular detail to design reduction schedules for patients, especially those with which has led to the use of informal guidance such as The Ashton Manual [31].

Further research is therefore needed into the most effective ways to stop these drugs, especially for different individuals. For example, there are no studies examining how to safely stop z-drugs or gabapentinoids [21]. Most studies of antidepressant discontinuation are aimed at examining their relapse prevention properties and have used short tapering periods. For example, a systematic review of approaches to stop antidepressants found most tapering regimens lasted four weeks or less and conclusions could not be drawn about the most effective and safe way to stop this medication [43]. An exhaustive NICE review on this topic undertaken to develop national guidance concluded that most studies of dose reduction of these drug types “did not reflect clinical practice.” [21]. There are currently two trials taking place examining approaches to help people safely stop antidepressants–the REDUCE trial in the UK [44] and the RELEASE trial in Australia [45].

Shared decision making and an individualised, flexible approach to withdrawal is in line with new clinical guidelines, such as NICE who promote shared decision making as both an ethical and legal requirement [21, 46].

One of the common practices in the deprescribing services was to provide psychosocial support. This is in line with research findings, where the provision of psychosocial support during withdrawal, such as cognitive behavioural therapy (CBT), relaxation, strategies to address insomnia, and psychoeducation, have been found to increase discontinuation rates and potentially reduce the chances of experiencing a relapse [37, 42, 47]. Although this is under-researched with some conflicting evidence [48].

Participants reported that there was a lack of awareness of withdrawal by some clinicians, leading patients to seek help from deprescribing services. This lack of awareness may arise from minimisation of the incidence, severity and duration of withdrawal effects by drug manufacturers, and academics, instead emphasising the risk of relapse [17, 49] reflected in the official NICE guidance until recently (which described ‘discontinuation symptoms as mild and self-limiting’) [50]. Our research supports other findings [51] that there has been widespread messaging about ‘chemical imbalances’ underlying mental health conditions, leading clinicians and patients to believe that medication is required to ameliorate these deficiencies [52, 53]. As a consequence, doctors may be primed to see relapse, and not withdrawal, leading to widespread misdiagnosis. It is hoped that recent updates to guidelines will start to update clinical practice [21].

### Strengths and limitations

This is the first international survey of deprescribing services, finding that practice is largely similar across different contexts and countries, largely arrived at by organic development of services. Our diverse study team of clinicians, researchers and those with lived experience gave a range of perspectives, informed our methods, enabled access to important networks, and provided a richer understanding of the topic and related practices. Our survey was conducted in English with recruitment through English-language networks which may have limited access to non-UK services. Our connection to an established global network of clinicians involved in deprescribing is a strength of our recruitment strategy, although it is possible less well connected services may not have been found by our strategy. Due to funding restrictions this was a rapid project which may have limited the depth of our qualitative analysis. The use of full transcripts instead of selected transcription could have given a better understanding of the nuances of the services [28, 54]. Although coding data directly from audio recordings ensures that nonverbal information such as intonation are retained, which can often be lost in full transcription [54].

## Conclusion

The significant human [1] and economic cost [4] of prescribed drug dependence and withdrawal have been documented and there is a need to establish deprescribing services to fill the gap in the current health system. We have summarised the common practices of existing deprescribing services and hope that the NHS commissioning framework captures these elements alongside the new NICE withdrawal guidance [21]. There is an urgent need for patients to have deprescribing services established not only for patients already experiencing problems, but for all those soon to be identified by initiatives such as the Structured Medication Review programme. Deprescribing services would reduce the harm caused to patients by unnecessary continuation of medication, and the harm produced by inexpert discontinuation of medication as well as the cost in terms of lost productivity and excess demand on the health system.

## Supporting information

S1 File Supplement 1 (S1) Appendix: Full survey questions; Supplement 2 (S2) Appendix: consolidated criteria for reporting qualitative studies (COREQ): 32-item checklist [24].
(DOCX)
