# Supplementary material for: “Stabilise-reduce, stabilise-reduce”: A survey of the common practices of deprescribing services and recommendations for future services
Source: PLoS One. 2023 Mar 15;18(3):e0282988. doi: 10.1371/journal.pone.0282988 (PMC10016688; doi:10.1371/journal.pone.0282988)
Supplement: S1 File — Supplement 1 (S1) Appendix: Full survey questions; Supplement 2 (S2) Appendix: consolidated criteria for reporting qualitative studies (COREQ): 32-item checklist [24]. (DOCX) [file pone.0282988.s001.docx]

**Supplement**

**Supplement 1 (S1) Appendix**

Full survey questions

**Think about the service pre-COVID-19, will ask at the end about changes due to COVID-19**

**About the organisation/service**

1. Could you start by telling me a bit about you and your role – how long have you been in this role?
2. When were you set up?
3. Legal form: Charity/CIC/NHS or other health org service/for-profit
4. How many staff/what mix of paid/volunteer/disciplines? Full or part-time
5. What’s your catchment area? Do you know the population? Can you estimate the number of potential beneficiaries in your area?
6. What’s your turnover/annual budget? Do you know the cost per head of your service?
7. Who funds the service? What obligations do you have in order to secure continuing funding? (e.g. reporting/monitoring, re-application for funds) If you charge per patients, what are the charges?

**About the service/support you offer**

1. Can you describe the range of services/supports that you offer? Who provides this? e.g. professional background, lived experience (LX). Can you describe a typical support package someone might get? E.g. drug effect information, pharmacy/prescriber support, 1:1, group, face to face or phone support, peer support
2. How do you assess each person’s support needs and plan to meet them? How much say does each person have in what support they get?
3. Do you offer alternatives to medication, either to help people through withdrawal or to enable them to deal with their original problem without medication?
4. Who provides these? paid/volunteer/professional background/LX background?
5. Do you have specific approaches to help people with physical/psychological effects of meds withdrawal?
6. How do you deal with any crisis or setbacks in someone’s progress? E.g. extreme withdrawal reaction, difficulties with re-emergence of presenting problems. Is there any support available outside of usual working hours? If someone became suicidal do you manage this within your service or would you refer them to regular mental health services?
7. Do you support people with protracted withdrawal symptoms after coming off their medications?
8. What’s the typical length of time you support someone? Are there time limits on support? Are people offered aftercare or directed to further support once their time with you ends?
9. What other support or strategies do people use/have outside the service (e.g. family support, exercise, lifestyle changes- diet, sleep, online support groups)?

**For services that provide prescribing/tapering support**

1. Do you offer specific tapering methods? What are these? Are they written down? What’s the typical length of a tapering programme, and what factors do you take into account when deciding this?
2. How did you develop your tapering methods (e.g. guidelines).
3. Do you ask GPs to provide prescriptions for the patient or does your service provide this?
4. Do you provide or suggest liquid preparations? Or suggest patients open capsules to count out beads, or grind up tablets to weigh them with a jeweller’s scale or make liquid suspensions to be used with syringes? Do you ever recommend tapering strips? What other means of physically titrating medications do you recommend/suggest?
5. Do you switch people to different versions of the same medication class or recommend this be done? E.g. longer-acting formulations.
6. How do you vary tapering methods to the individual? How do you monitor withdrawal effects? Are there specific metrics? If so, how do you get them to monitor these symptoms e.g. diary/form/spreadsheet?

**Outcomes**

1. How would you describe a successful outcome for someone?
2. What outcomes measures do you record/report? E.g. quality of life, anxiety or depression scores? Measures of insomnia? Side effects? Functioning, PHQ9/GAD7. If outcomes aren’t available online, are there any you could share with us?
3. Do you have targets you need to meet? How are you doing with these?
4. Do you gather feedback from your beneficiaries? Can you share any of this?
5. How do you measure adverse outcomes (e.g. someone becoming unwell after withdrawing, protracted withdrawal).

**About development of the service**

1. How/why did the service come into being?
2. Were there any issues in setting up the service?
3. What development work was needed to set the service up? Who was involved in this? E.g. LX, practitioner, commissioner
4. Did you consider a range of options for the service? What made you select the one/s you did?
5. How long did service development take?

**About leadership/beneficiary leadership**

1. Who leads the service? E.g. psychiatrist/psychologist/other professionals/beneficiaries/a mixture
2. How much involvement of beneficiaries is there in: day-to-day running?; longer term decision-making
3. Can you describe any beneficiary involvement mechanisms you have? What specific impacts have involvement had on the way your service operates?
4. Do you employ peer workers/people with LX - are they able to use this experience openly?
5. If you employ those with personal experience, is this written into person specifications as desirable/essential?
6. To what extent do you think lived experience is important in the creation of future deprescribing services?

**Coming into contact with the service**

1. Do patients ask to come to the service or is it more common that their healthcare professional suggests that they go?
2. What proportion are self-referred or instigate the referral themselves (if this is known).
3. Where do referrals come from? Self or service/staff/professional referral? What’s the referral process? If a mixture of referral sources do you have data on the proportions? Are any potential sources of referrals conspicuously present/absent?
4. How would professional referrers (e.g. GPs, psychiatrists) know about your service (do you give out leaflets, give talks?)?
5. Do you have specific referral criteria/exclusion criteria (e.g. type or dose of drug, duration of use, unwanted medication, unnecessary’ medication defined (i.e. antidepressants for more than a year, benzodiazepines for more than one month?).
6. How many referrals do you get yearly? Are you able to deal with the level of demand?
7. What are the drugs that people are referred for (breakdown by proportion if available)? Do you help people come off gabapentinoids (pregabalin, gabapentin)?
8. If you have GPs/drug workers in GP practices/ asking people if they want to use the service, what proportion of those patients will want to access your service? What are the main reasons they do not want to (think the medications are helpful/think that coming off will be too hard)?
9. Do you have priorities for who you provide services to? E.g. pregnant, older people, other high-risk groups?
10. What factors affect the number of referrals you get? E.g. lack of knowledge/awareness/any barriers

**About the people the service/org supports**

1. How many people do you support in a year?
2. Do you have any demographic data you can share? Do you see people under 18?
3. What range of medications will people have been taking? Multiple medications? Typical prescribing duration?
4. How do people describe their reasons for attending? E.g. “sent” by a GP, tried to come off previously, want better quality of life, adverse effects from drugs
5. What reasons do people describe for originally being prescribed the medication? Mental health? Physical health? Do people generally believe their prescriptions to have been appropriate
6. What sort of complicating factors might people experience? Eg recreational drugs, other health conditions, demographic/protected characteristics/S-E status
7. What are people’s main worries when they start reducing their medication?

**Raising awareness**

1. How would you describe the level of awareness of withdrawal issues amongst - Health and social care professionals (and subgroups e.g. primary/secondary care)? The general public? Has this improved over the last couple of years, or not?
2. Do you provide any training/education to raise awareness of medication withdrawal? Does this include education about the effectiveness of these medications?
3. Do you conduct specific awareness training/education about your service e.g. to professional or LX groups?
4. How supportive/unsupportive do you find wider institutions or networks in terms of your service?

**The future and aspirations**

1. Do you have ideas for further development of your service?
2. Are there any threats to your service?
3. What single thing would make the biggest difference to supporting medication withdrawal? (blue sky/unlimited budget).

**Covid impact**

1. How has the service been effected/changed by COVID-19?

**Supplement 2 (S2) Appendix**

Consolidated criteria for reporting qualitative studies (COREQ): 32-item checklist [1]

| **No** | **Item** | **Guide questions/description** | **Reported on page no** | **Comment if not reported in manuscript or further comments** |
| --- | --- | --- | --- | --- |
| **Domain 1: Research team and reflexivity** | | | |  |
| Personal Characteristics | | | |  |
| 1 | Interviewer/facilitator | Which author/s conducted the interview or focus group? | 9 | - |
| 2 | Credentials | What were the researcher’s credentials? E.g. PhD, MD | NA | Ruth E Cooper: PhD  Michael Ashman: MPH  Jo Lomani: MSc  Joanna Moncrieff: MD  Anne Guy: PsychD  James Davies: PhD  Nicola Morant: PhD  Mark Horowitz: PhD |
| 3 | Occupation | What was their occupation at the time of the study? | 9 | - |
| 4 | Gender | Was the researcher male or female? | Title page, 9 | - |
| 5 | Experience and training | What experience or training did the researcher have? | 9 | - |
| Relationship with participants | | | |  |
| 6 | Relationship established | Was a relationship established prior to study commencement? | 10 | The lived experience researcher MA, knew one participant prior to interview in a professional capacity. MH (who conducted two interviews with MA) knew one participant in a professional capacity prior to interview. |
| 7 | Participant knowledge of the interviewer | What did the participants know about the researcher? e.g. personal goals, reasons for doing the research | NA | See above (Q6) for prior relationships with interviewers.  To enhance rapport and a relationship built on mutual understanding, the lived experience researcher MA, discussed his lived experience with participants where appropriate. |
| 8 | Interviewer characteristics | What characteristics were reported about the interviewer/facilitator? e.g. Bias, assumptions, reasons and interests in the research topic | NA | To enhance rapport and a relationship built on mutual understanding, the lived experience researcher MA, discussed his lived experience with participants where appropriate. |
| **Domain 2: study design** | | | |  |
| Theoretical framework | | | |  |
| 9 | Methodological orientation and Theory | What methodological orientation was stated to underpin the study? e.g. grounded theory, discourse analysis, ethnography, phenomenology, content analysis | 11 | **-** |
| Participant selection | | | |  |
| 10 | Sampling | How were participants selected? e.g. purposive, convenience, consecutive, snowball | 10 | - |
| 11 | Method of approach | How were participants approached? e.g. face-to-face, telephone, mail, email | 10 | - |
| 12 | Sample size | How many participants were in the study? | 13 | - |
| 13 | Non-participation | How many people refused to participate or dropped out? Reasons? | 10 | - |
| Setting | | | |  |
| 14 | Setting of data collection | Where was the data collected? e.g. home, clinic, workplace | 10 | - |
| 15 | Presence of non-participants | Was anyone else present besides the participants and researchers? | 10 | Only participants and researcher/s present |
| 16 | Description of sample | What are the important characteristics of the sample? e.g. demographic data, date | Table 1 | Our sample characteristics are the service characteristics in Table 1. The number of participants interviewed and their role in the service are also detailed in Table 1. |
| Data collection | | | |  |
| 17 | Interview guide | Were questions, prompts, guides provided by the authors? Was it pilot tested? | 9-10, Supplement 1 (S1) appendix | - |
| 18 | Repeat interviews | Were repeat interviews carried out? If yes, how many? | NA | No repeat interviews were carried out |
| 19 | Audio/visual recording | Did the research use audio or visual recording to collect the data? | 10 | - |
| 20 | Field notes | Were field notes made during and/or after the interview or focus group? | 11 | - |
| 21 | Duration | What was the duration of the interviews or focus group? | 13 | **-** |
| 22 | Data saturation | Was data saturation discussed? | **-** | This was a rapid project due to funder requirements with project set-up, data collection and analysis restricted to 3 months (Jan-March 2021). We recruited as many services as we could, within this timeframe. |
| 23 | Transcripts returned | Were transcripts returned to participants for comment and/or correction? | 13 | Due to the time limited nature of the project we conducted a rapid form of framework analysis with selected transcription. Selected transcripts were not returned to participants for comment/correction. We instead sent the manuscript to participants for comment/correction |
| **Domain 3: analysis and findings** | | | |  |
| Data analysis | | | |  |
| 24 | Number of data coders | How many data coders coded the data? | 11-13 | **-** |
| 25 | Description of the coding tree | Did authors provide a description of the coding tree? | 11-13 | **-** |
| 26 | Derivation of themes | Were themes identified in advance or derived from the data? | 11-13 | Themes were derived from the data |
| 27 | Software | What software, if applicable, was used to manage the data? | 11-13 | **-** |
| 28 | Participant checking | Did participants provide feedback on the findings? | 13 | - |
| Reporting | | | | |
| 29 | Quotations presented | Were participant quotations presented to illustrate the themes / findings? Was each quotation identified? e.g. participant number | 31-40, Table 3 | **-** |
| 30 | Data and findings consistent | Was there consistency between the data presented and the findings? | **-** | Data and findings are consistent throughout the manuscript. |
| 31 | Clarity of major themes | Were major themes clearly presented in the findings? | 31-40, Table 3 | **-** |
| 32 | Clarity of minor themes | Is there a description of diverse cases or discussion of minor themes? | 31-40, Table 3 | **-** |

**References**

1. Tong A, Sainsbury P, Craig J. Consolidated criteria for reporting qualitative research (COREQ): a. Int J Qual Heal Care. 2007;19: 349–357. doi:10.1093/intqhc/mzm042
